# Timing for initiation of continuous renal replacement therapy in patients with septic shock and acute kidney injury

**DOI:** 10.1186/cc10968

**Published:** 2012-03-20

**Authors:** HP Shum, KC Chan, MC Kwan, WT Yeung, WS Cheung, WW Yan

**Affiliations:** 1 Pamela Youde Nethersole Eastern Hospital, Hong Kong

## Introduction

The optimal timing for initiation of renal replacement therapy (RRT) in septic acute kidney injury (AKI) remains controversial. The aim of this study is to investigate the impact of early versus late initiation of continuous RRT (CRRT), as defined using the simplified RIFLE classification, on organ dysfunction among patients with septic shock and AKI.

## Methods

Patients were divided into early (sRIFLE Risk) or late (sRIFLE Injury or Failure) initiation of RRT. Patients with chronic kidney disease stage 5 or on dialysis were excluded.

## Results

One hundred and twenty patients admitted within a 3.5-year period fulfilled inclusion criteria. Thirty-one (26%) underwent early, 89 (74%) had late CRRT. No significant difference was noted between the two groups with respect to change in total SOFA score/non-renal SOFA score in the first 24/48 hours after initiation of CRRT, vasopressor use, dialysis requirement and mortality (at 28 days, 3 months and 6 months). The change of nonrenal SOFA score 48 hours after CRRT correlated with the SOFA score at the start of CRRT (*P *= 0.034) and the APACHE IV risk of death (*P *= 0.000), but not the glomerular filtration rate (GFR) at the start of CRRT (*P *= 0.348). See Tables 1 and 2.

**Table 1:** Baseline characteristics and parameters on initiation of CRRT

	Early	Late	*P *value
Age	70.7 ± 15.1	69.3 ± 13.1	0.614
APACHE IV	119 ± 31	131 ± 37	0.110
Starting GFR	36.2 ± 20.9	181 ± 8.2	< 0.001
Start SOFA	11.6 ± 3.3	133 ± 2.7	0.006

**Table 2:** Outcome parameters

	Early	Late	*P *value
NR SOFA 0 to 48	-0.52 ± 3.91	-0.71 ± 3.57	0.827
Hospital death	17 (54.8%)	48 (53.9%)	0.931
28-day survival	16 (51.6%)	46 (51.7%)	0.994

## Conclusion

For septic shock with AKI, no significant difference in organ function and outcome was noted when the timing of initiation of CRRT was classified using sRIFLE criteria. Subsequent improvement of organ function correlated with initial SOFA and APACHE scores instead of the GFR (which determine sRIFLE class) on starting of CRRT. The use of more global assessment tools, such as the SOFA score, for stratification purposes on appropriate timing of CRRT warrants further investigation.

